# A Nomogram for Preoperative Prediction of Tumor Aggressiveness and Lymphovascular Space Involvement in Patients with Endometrial Cancer

**DOI:** 10.3390/jcm14113914

**Published:** 2025-06-02

**Authors:** Riccardo Valletta, Giacomo Avesani, Vincenzo Vingiani, Bernardo Proner, Martin Steinkasserer, Sara Notaro, Francesca Vanzo, Giovanni Negri, Caterina Vercelli, Matteo Bonatti

**Affiliations:** 1 Department of Radiology, Hospital of Bolzano (SABES-ASDAA), Teaching Hospital of Paracelsius Medical University (PMU), 39100 Bolzano-Bozen, Italy; riccardo.valletta@gmail.com (R.V.); vinc.vingia@yahoo.it (V.V.); bernardo.proner@sabes.it (B.P.); 2 Department of Imaging and Radiotherapy, Fondazione Policlinico Universitario Agostino Gemelli IRCCS, 00168 Rome, Italy; giacomo.avesani@policlinicogemelli.it; 3 Department of Ginecology, Hospital of Bolzano (SABES-ASDAA), Teaching Hospital of Paracelsius Medical University (PMU), 39100 Bolzano-Bozen, Italy; martin.steinkasserer@sabes.it (M.S.); sara.notaro@sabes.it (S.N.); francesca.vanzo@sabes.it (F.V.); 4 Department of Pathology, Hospital of Bolzano (SABES-ASDAA), Teaching Hospital of Paracelsius Medical University (PMU), 39100 Bolzano-Bozen, Italy; giovanni.negri@sabes.it; 5 Department of Health Sciences (DISSAL), University of Genoa, 16126 Genoa, Italy; catevercelli@gmail.com

**Keywords:** uterus, endometrial neoplasms, lymphovascular space invasion, neoplasm grading, magnetic resonance imaging, nomogram model

## Abstract

**Background/Objectives:** To develop a nomogram for predicting tumor aggressiveness and the presence of lymphovascular space involvement (LVSI) in patients with endometrial cancer (EC) using preoperative MRI and pathology–laboratory data. **Methods:** This IRB-approved, retrospective, multicenter study included 245 patients with histologically confirmed EC who underwent preoperative MRI and surgery at participating institutions between January 2020 and December 2024. Tumor type and grade, both from preoperative biopsy and surgical specimens, as well as preoperative CA125 and HE4 levels, were retrieved from institutional databases. A preoperative MRI was used to assess tumor morphology (polypoid vs. infiltrative), maximum diameter, presence and depth (< or >50%) of myometrial invasion, cervical stromal invasion (yes/no), and minimal tumor-to-serosa distance. The EC-to-uterus volume ratio was also calculated. **Results:** Among the 245 patients, 27% demonstrated substantial LVSI, and 35% were classified as aggressive on final histopathology. Multivariate analysis identified independent MRI predictors of LVSI, including cervical stromal invasion (OR = 9.06; *p* = 0.0002), tumor infiltration depth (OR = 2.09; *p* = 0.0391), and minimal tumor-to-serosa distance (OR = 0.81; *p* = 0.0028). The LVSI prediction model yielded an AUC of 0.834, with an overall accuracy of 78.4%, specificity of 92.2%, and sensitivity of 43.1%. For tumor aggressiveness prediction, significant predictors included biopsy grade (OR = 8.92; *p* < 0.0001), histological subtype (OR = 12.02; *p* = 0.0021), and MRI-detected serosal involvement (OR = 14.39; *p* = 0.0268). This model achieved an AUC of 0.932, with an accuracy of 87.0%, sensitivity of 79.8%, and specificity of 91.2%. Both models showed excellent calibration (Hosmer–Lemeshow *p* > 0.86). **Conclusions:** The integration of MRI-derived morphological and quantitative features with clinical and histopathological data allows for effective preoperative risk stratification in endometrial cancer. The two nomograms developed for predicting LVSI and tumor aggressiveness demonstrated high diagnostic performance and may support individualized surgical planning and decision-making regarding adjuvant therapy. These models are practical, reproducible, and easily applicable in standard clinical settings without the need for radiomics software, representing a step toward more personalized gynecologic oncology.

## 1. Introduction

Endometrial cancer (EC) is the second most common gynecologic malignancy worldwide, and its incidence is progressively increasing, particularly in industrialized countries [1,2,3]. According to the International Federation of Gynecology and Obstetrics (FIGO) guidelines, EC staging is surgical, but MRI plays a pivotal role in optimizing treatment strategies [4,5]. The recently updated FIGO staging system integrated anatomic findings with pathologic and molecular variables to improve patients’ prognostic stratification [4]. According to the 2023 update, tumor histological type and grade definition, which enables the subdivision of ECs into aggressive (G3 endometrioid or non-endometrioid tumors) and non-aggressive (G1-G2 endometrioid) subtypes, as well as the presence of substantial lymphovascular space invasion (LVSI) assessment, are crucial to correctly stage EC. However, discrepancies exist in tumor type and grade assessment between preoperative biopsy and surgical specimen, and LVSI can be addressed on the surgical specimen only [6,7,8,9,10]. Consequently, a definite preoperative MRI staging is often impossible [11].

On the other hand, many papers explored the potential role of MRI as an additional tool for assessing ECs’ biological behavior. Many MRI features (e.g., the presence of deep myometrial infiltration, large tumor size, high tumor/uterus volume ratio, and low ADC values) have been variably associated with high-grade neoplasms and with the presence of LVSI [6,8,12,13,14,15,16,17,18,19]. The first-line treatment for endometrial cancer (EC) is total hysterectomy with bilateral salpingo-oophorectomy and nodal staging that may be performed using a sentinel node biopsy (SLNB) to reduce morbidity without compromising oncological outcomes, particularly in low-risk cases [20].

A key factor guiding both treatment decisions and prognostic stratification is the presence of a substantial lymphovascular space invasion (LVSI). LVSI is considered an early marker of lymphatic metastatic spread and is strongly correlated with lymph node metastases, pelvic recurrence, and decreased overall survival [20,21,22]. Nonetheless, its detection is generally based on a final histopathological evaluation.

Accurately identifying LVSI in the preoperative setting—ideally through MRI—could significantly improve risk classification and inform the need for more extensive surgical intervention and/or adjuvant therapies such as radiotherapy or chemotherapy.

The aim of our study was to identify MRI parameters that can be incorporated into the development of a nomogram capable of predicting tumor aggressiveness and the potential presence of LVSI in endometrial cancer.

## 2. Materials and Methods

Study population

The Institutional Review Board approved our multicenter retrospective study; the requirement for informed consent was waived. During the period January 2020–December 2024, 273 consecutive patients affected by histologically proven EC who underwent preoperative pelvic MRI and surgery at the two participating Institutions (Central Hospital, Bolzano, Italy, and IRCSS Policlinico Agostino Gemelli, Rome, Italy) were considered for inclusion. Inclusion criteria were the availability of a preoperative pelvic MRI scan (within 4 weeks before surgery) in patients who did not receive any treatment before the scan (endometrial ablation, neoadjuvant chemoradiotherapy, etc.). Exclusion criteria were non-accessible data from the institutional database (3/280, 1%), the time between MRI and surgery >30 days (10/245, 4%), and the presence of motion or ferromagnetic artifacts (15/280, 6%). Therefore, our patient population encompassed 245 women with a median age of 66 years (range 58–74 years).

MRI protocol

All MR examinations were performed on 1.5 T MRI scanners (Ingenia, Philips, Best, Netherlands, or Signa Excite; GE Healthcare, Little Chalfont, UK) with the patient lying supine on the table, using multi-channel phased-array body coils. The patient was asked to fast for 6 h before the examination and to void 1 h before it; moreover, 20 mg of butylscopolamine bromide (Buscopan, Boehringer Ingelheim, Ingelheim, Germany) was administered intramuscularly just before the beginning of the examination.

MRI pulse sequences and image parameters are reported in Table 1.

High-resolution T2-weighted images (T2-WI) and contrast-enhanced T1-weighted images (CE T1-WI) were acquired along three orthogonal planes (para-sagittal, para-axial, and para-coronal), according to the endometrial cavity’s longest axis, whereas diffusion-weighted images (DWI) were acquired on two planes only (para-axial and para-sagittal); apparent diffusion coefficient (ADC) maps were generated from isotropic diffusion-weighted images. CE T1-WI was acquired after an intravenous bolus injection of 0.1 mmolGd/kg of paramagnetic contrast material, followed by a 20 mL saline flush.

Image analysis

Image analysis was performed by one radiologist (10 years of experience in pelvic MRI) on a commercially available workstation. The reader was aware of the presence of a histologically proven EC but was unaware of surgical and histological findings.

Qualitative image analysis included the lesion’s growth pattern (polypoid or infiltrative), the presence of myometrial invasion (yes or no), the presence of myometrial infiltration exceeding 50% of its thickness (yes or no), the presence of cervical stromal invasion (yes or no), the presence of serosal or subserosal involvement (yes or no), the presence of tubaric or adnexal involvement (yes or no), the presence of parametrial involvement (yes or no), the presence of vaginal involvement (yes or no), the presence of bladder or rectal involvement (yes or no), the presence of pelvic nodal involvement (yes or no), and the presence of pelvic peritoneal carcinosis (yes or no).

Quantitative image analysis included both EC and uterus three orthogonal diameters measurements, and EC-to-serosa minimal distance measurement. EC’s maximum diameter was annotated. EC and uterus volumes were estimated using the ellipsoid formula; the EC/uterus volume ratio was calculated.

Histological and laboratory data

Histological data were retrieved from institutional databases, including EC’s histological type and grade, both in a preoperative biopsy and on the surgical specimen, and the presence of substantial LVSI on the surgical specimen. G1-G2 endometrioid ECs were considered non-aggressive, whereas G3 endometrioid and non-endometrioid ECs were considered aggressive. Laboratory data could not be incorporated into the models due to their limited availability. LVSI was considered substantial if a diffuse or multifocal LVSI (3 or more involved vessels) was recognized around the tumor or a massive LVSI was recognized in the myometrium with a spray-like growth, regardless of the degree of myometrial invasion [23].

Statistical analysis

MedCalc (version 20, MedCalc Software) was used for all statistical analyses. RStudio (Version 2024.12.1+563) was used to generate the nomogram. Continuous variables are expressed as mean ± SD or as median with interquartile range (IQR), depending on their distribution. Normality was assessed using the D’Agostino–Pearson test. Both univariate and multivariate analyses were performed to evaluate the influence of various independent variables on the probability of LVSI and aggressiveness. An independent-sample two-tailed t-test or the Mann–Whitney U-test was applied, as appropriate, to compare continuous variables, while Fisher’s exact test and the chi-square test were used for dichotomous variables. Variables with *p*-values < 0.10 in the univariate analysis were considered for inclusion in the multivariate logistic regression analysis. However, for the LVSI model, variables indicative of advanced disease (e.g., serosal involvement, adnexal or parametrial invasion, vaginal involvement, bladder or rectal extension, lymph node involvement, distant metastases, and peritoneal carcinomatosis) were excluded, as they are not typically available in early or operable disease. The same exclusion criteria were applied to the tumor aggressiveness model.

The final set of predictors included in the multivariate models was determined based on both statistical significance and clinical feasibility, prioritizing parameters obtainable preoperatively through standard MRI and endometrial biopsy. Model calibration was assessed using the Hosmer–Lemeshow test. Each variable’s contribution to the model was weighted according to its regression coefficient (β), representing the strength and direction of its association with the outcome. These coefficients were then proportionally scaled to assign individual point values in the nomogram. The variable with the greatest impact on the outcome received the highest weight, and all other variables were adjusted relative to it. The nomogram thus functions as a graphical representation of the logistic regression model, enabling the calculation of individualized risk probabilities by summing the weighted contributions of each predictor.

A *p*-value < 0.05 was considered statistically significant.

## 3. Results

For the preoperative biopsy, the pathologist classified 207/245 (84%) neoplasms as endometrioid and 38/245 (16%) as non-endometrioid (15/38 serous type, 3/38 clear cell type, and 20/38 as mixed type). A total of 94/245 (38%) neoplasms were classified as grade 1, 81/245 (33%) as grade 2, and 70/245 (29%) as grade 3. Consequently, 171/245 (70%) ECs were considered non-aggressive, and 74/245 (30%) were considered aggressive.

For the MRI, the growth pattern was defined as polypoid in 168/245 (69%) cases and infiltrative in 77/245 (31%). A myometrial invasion was present in 204/245 (83%) of the cases, and it was >50% in 86/245 (17%). A cervical stromal invasion was present in 25/245 (10%) cases, there was serosal or subserosal involvement in 13/245 (5%), tubaric or adnexal involvement in 5/245 (2%), parametrial involvement in 5/245 (2%), vaginal involvement in 3/245 (1%), bladder or rectal involvement in 0/245 (0%), pelvic nodal involvement in 21/245 (9%), and pelvic peritoneal carcinosis in 3/245 (1%).

On the surgical specimen, the pathologist classified 206/245 (84%) neoplasms as endometrioid and 39/245 (16%) as non-endometrioid. A total of 48/245 (20%) neoplasms were classified as grade 1, 113/245 (46%) as grade 2, and 84/245 (34%) as grade 3. In 71 cases, a tumor grade upgrade was observed between the biopsy and the final histology (mostly from G1 to G2 or G3), whereas 16 cases showed a downgrade. Consequently, 159/245 (65%) ECs were considered non-aggressive, and 86/245 (35%) were considered aggressive. A substantial LVSI was present in 66/245 (27%) cases and absent in 179/245 (73%).

The results of the univariate analysis comparing patients with and without LVSI are reported in Table 2.

In 22/245 (9.0%) cases, the tumor classification changed from non-aggressive to aggressive between the biopsy and the surgical specimen, while in 10/245 (4.1%), the opposite occurred.

Among the predictors included in the logistic regression analysis, MRI-detected cervical involvement significantly increased the odds of LVSI positivity (OR = 8.89, 95% CI: 2.79–28.31, *p* = 0.0002). In contrast, a greater minimal tumor-to-serosa distance was associated with a lower likelihood of LVSI (OR = 0.80, 95% CI: 0.70–0.93, *p* = 0.0024). Additionally, tumor infiltration depth was a significant predictor (OR = 2.11, 95% CI: 1.05–4.24, *p* = 0.0360). The model demonstrates good discriminative ability, with an AUC of 0.834 (95% CI: 0.780–0.880) and an overall classification accuracy of 79.65%. However, the sensitivity remains relatively low (44.62%). In contrast, specificity was high (93.37%), meaning that the model effectively excludes LVSI-negative cases. The Hosmer–Lemeshow test yielded a chi-squared value of 2.87 (DF = 8, *p* = 0.942), indicating excellent model calibration and no significant deviation between observed and predicted values.

**Figure 1:**
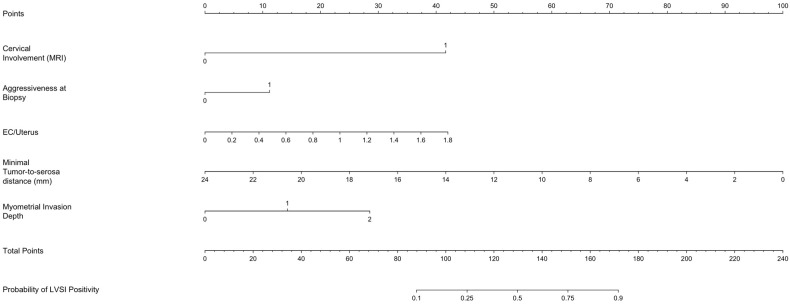
Nomogram for predicting the probability of lymphovascular space invasion (LVSI) on definitive pathological specimen in patients with endometrial cancer, based on logistic regression analysis. To use the nomogram, locate the patient-specific value for each variable on its corresponding axis, draw a vertical line up to the “Points” axis to determine the individual score, then sum all the points and find the total on the “Total Points” axis. Finally, draw a vertical line downward to determine the predicted probability at the bottom of the chart. This graphical tool enables intuitive, individualized risk assessment using routinely available clinical and imaging data. The contribution of each variable is weighted according to its odds ratio in the logistic regression model.

Figure 1 presents a nomogram developed based on the logistic regression model to predict the probability of LVSI positivity. The nomogram integrates significant predictors, allowing for individualized risk estimation. Each predictor contributes a specific number of points, which are summed to determine the overall probability of LVSI. This graphical tool enhances clinical decision-making by providing an intuitive risk assessment based on the model’s findings.

The results of the univariate analysis comparing patients with and without aggressive histotypes are reported in Table 3.

Among the predictors included in the logistic regression analysis, biopsy grade (OR = 8.92, 95% CI: 4.56–17.46, *p* < 0.0001) and histology (OR = 12.02, 95% CI: 2.47–58.57, *p* = 0.0021) significantly increased the odds of tumor aggressiveness. Additionally, serosal involvement on MRI was strongly associated with tumor aggressiveness (OR = 14.39, 95% CI: 1.36–152.31, *p* = 0.0268). Moreover, higher EC/uterus values were significantly associated with increased tumor aggressiveness (*p* = 0.0089). The model demonstrates excellent discriminative ability, with an AUC of 0.932 (95% CI: 0.891–0.961) and an overall classification accuracy of 87.01%. Sensitivity and specificity were 79.76% and 91.16%, respectively. The Hosmer–Lemeshow test yielded a chi-squared value of 3.87 (DF = 8, *p* = 0.8688), confirming excellent model calibration.

**Figure 2:**
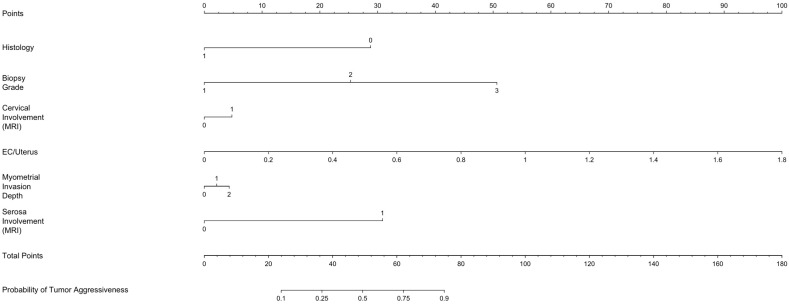
Nomogram for predicting the probability of tumor aggressiveness in endometrial cancer based on the logistic regression model. To use the nomogram, locate the patient-specific value for each variable on its corresponding axis, draw a vertical line up to the “Points” axis to determine the individual score, then sum all the points and find the total on the “Total Points” axis. Finally, draw a vertical line downward to determine the predicted probability at the bottom of the chart. This graphical tool enables intuitive, individualized risk assessment using routinely available clinical and imaging data. The contribution of each variable is weighted according to its odds ratio in the logistic regression model.

Figure 2 presents a nomogram developed based on the logistic regression model to predict the probability of tumor aggressiveness. The nomogram integrates significant predictors, allowing for individualized risk estimation. Each predictor contributes a specific number of points, which are summed to determine the overall probability of tumor aggressiveness. This graphical tool enhances clinical decision-making by providing an intuitive risk assessment based on the model’s findings.

## 4. Discussion

In this multicenter retrospective study, we developed two predictive nomograms combining clinical–pathological and conventional MRI features to estimate, prior to surgery, the likelihood of tumor aggressiveness and lymphovascular space invasion (LVSI) in patients with endometrial cancer. The analysis included 245 women who underwent preoperative pelvic MRI and surgery, with evaluation of tumor morphology, volume-related parameters, and biopsy-derived histological data.

One of the most critical aspects in the management of endometrial cancer is the reliability of endometrial biopsy in accurately determining tumor aggressiveness. In our cohort, we found a discrepancy between biopsy and surgical specimen histology in 86 out of 245 cases (35%), confirming a substantial preoperative instability in histotype classification. We observed 71 cases of tumor grade upgrade between biopsy and final histology (mostly from G1 to G2 or G3), with 16 cases of downgrade, which may lead to an oncologically insufficient surgical procedure. In 22 cases, the tumor transitioned from non-aggressive to aggressive between the biopsy and the surgical specimen. This finding is consistent with previous studies [24,25,26,27] and underlines the limitations of biopsy-based histotype determination due to tumor heterogeneity and sampling bias. As a result, there is often a systematic underestimation of tumor aggressiveness in the preoperative setting. Such discrepancies may directly impact surgical planning and decisions regarding lymphadenectomy or adjuvant treatment, thus supporting the development of radiological prediction models that are not solely dependent on biopsy findings.

To address this matter, we developed and validated two predictive nomograms based on MRI features and preoperative clinico-pathological data to estimate lymphovascular space invasion (LVSI) and tumor aggressiveness in patients with endometrial cancer. The results show that combining morphological and quantitative MRI parameters with histological data from endometrial biopsy enables effective preoperative risk stratification with high diagnostic performance (AUC = 0.834 for LVSI; AUC = 0.932 for tumor aggressiveness).

MRI variables significantly associated with greater tumor aggressiveness included deep myometrial invasion (>50%), cervical stromal involvement, uterine serosal invasion, an increased tumor-to-uterus volume ratio, and a reduced tumor-to-serosa distance, indicating its potential role as a predictive continuous variable. Histological variables significantly associated with tumor aggressiveness were grade 3 (G3) at biopsy and non-endometrioid histology. MRI features significantly associated with the presence of LVSI were cervical stromal invasion, deep myometrial invasion (>50%), a high tumor-to-uterus volume ratio, tumor infiltration depth, and a short tumor-to-serosa distance, suggesting that deeper tumor invasion increases the probability of LVSI. Histological predictors of LVSI included grade 3 and non-endometrioid histology on biopsy. These findings emphasize the ability of MRI-derived morphological features to provide reliable insights into tumor biology, particularly regarding vascular invasion and differentiation grade. The nomogram developed to predict tumor aggressiveness (defined as G3 or non-endometrioid histology) was built using seven variables: biopsy histotype, biopsy grade, cervical invasion, serosal involvement, tumor-to-uterus volume ratio, infiltration depth, and tumor-to-serosa distance. The model presented an AUC of 0.932 (95% CI: 0.891–0.961), with an overall accuracy of 87.0%, sensitivity of 79.8%, and specificity of 91.2%. Calibration was excellent according to the Hosmer–Lemeshow test (*p* = 0.8688), indicating a strong ability to correctly classify aggressive tumors. The LVSI nomogram was developed using six variables: biopsy grade, aggressive histotype on biopsy, cervical invasion (MRI), tumor-to-uterus volume ratio, tumor infiltration depth, and tumor-to-serosa distance. The model showed an AUC of 0.834 (95% CI: 0.779–0.879), with an accuracy of 78.4%, sensitivity of 43.1%, and specificity of 92.2%, and demonstrated excellent calibration (Hosmer–Lemeshow *p* = 0.9081). The low sensitivity reflects the clinical challenge of identifying LVSI preoperatively; however, the high specificity allows for reliable exclusion of negative cases, which can help guide conservative surgical approaches or avoid extended lymphadenectomy.

Our results are consistent with several previous studies that aimed to identify preoperative predictors of LVSI. Kim et al. [28] proposed a predictive score based on tumor diameter, depth of myometrial invasion, tumor grade, and cervical involvement, achieving an AUC of 0.839, with 74.1% sensitivity and 80.5% specificity. Similarly, Meydanli et al. [29] identified a combination of grade, tumor size, and percentage of myometrial invasion as predictive of LVSI, with an AUC of 0.90. Wang et al. [6] combined multiparametric MRI radiomics and clinical variables, achieving AUCs of 0.914 in the training cohort and 0.912 in the validation cohort for LVSI prediction. Although our model is based on similar variables, it has the advantage of being entirely radiological and is, therefore, independent from intraoperative findings or invasive indices.

Several studies have also developed radiomics-based nomograms; Luo et al. [26] achieved an AUC of 0.820 in the training cohort and 0.807 in the test set. Ma et al. [30] reported AUCs of 0.959 in training and 0.926 in validation. Although these results are comparable or even superior to ours, our nomograms, which are entirely based on MRI and histological features, present a practical advantage: they can be easily replicated in any MRI-equipped center and are simpler to implement, potentially making them more suitable for routine clinical settings without the need for advanced feature extraction software.

Regarding predictors of tumor aggressiveness, our findings confirm the association between histological grade, non-endometrioid histotype, and serosal involvement with aggressive tumors. These findings are in line with what was reported by Rafiee et al. [31], who observed strong correlations between tumor grade, depth of invasion, and LVSI. Another paper [27] showed that deeper myometrial invasion is strongly associated not only with LVSI but also with lymph node metastases, recurrence, and poorer survival.

Our findings regarding the ability to predict LVSI agree with those of previous literature. Kim et al. [28] identified deep myometrial invasion, tumor size, and cervical involvement as key predictors of LVSI. Radiomics studies [6,26,30] have demonstrated comparable or higher AUCs; however, their clinical implementation is often constrained by the need for complex image processing, specialized software, and standardized acquisition protocols. In contrast, our nomogram is derived from conventional MRI parameters and biopsy data readily available in standard clinical workflows, providing a practical and reproducible tool for real-world preoperative risk stratification.

The ability to predict LVSI and tumor aggressiveness before surgery could significantly improve preoperative management in EC patients, aiding in the selection for lymphadenectomy, sentinel node mapping, or adjuvant therapy. This is particularly relevant considering findings by Buechi et al. [32], who showed that the presence of LVSI significantly reduces the negative predictive value of sentinel lymph node mapping.

Although our models were developed using a multicenter cohort with good generalizability, external validation is an essential step before clinical application. Predictive performance may vary depending on MRI protocols and population characteristics.

Our study has some limitations; first, it is a retrospective study and may be subject to selection bias. Furthermore, laboratory data could not be incorporated into the models due to their limited availability, as well as molecular information that was unavailable in too many cases to be included in the analyses. In addition, the lack of a direct comparison with radiomics-based models limits our ability to draw firm conclusions. On the other hand, image standardization and blinded histological assessments remain among the strengths of the study. Future developments may include the integration of our nomograms with molecular biomarkers (such as MSI, p53, and L1CAM), molecular information, and selected radiomic features using explainable AI algorithms, with the aim of building more robust and personalized hybrid prediction models.

## Figures and Tables

**Table 1:** Magnetic resonance imaging protocol: pulse sequences and parameters. FS = fat-saturated; TSE = turbo spin echo; EPI = echo planar imaging.

Pulse Sequence	Scanning Plane	TR/TE (ms)	Voxel Size (mm)	FoV (mm)
FS T2-weighted TSE	Axial (pelvis)	7700/83	1.3 × 0.9 × 6.0	400
T1-weighted TSE	Axial (pelvis)	730/10	0.9 × 0.6 × 6.0	350
T2-weighted TSE	Para-sagittal, para-axial, para-coronal (uterus)	3200/82	0.5 × 0.5 × 4.0	250
EPI (b = 0, 500, 1000 s/mm^2^)	Para-sagittal, pasa-axial (uterus)	3100/98	2.0 × 1.0 × 5.0	250
Contrast-enhanced T1-weighted TSE	Para-sagittal, para-axial, para-coronal (uterus)	606/9.5	1.3 × 0.8 × 4.0	250
Dynamic contrast-enhanced MR perfusion (DCE; optional sequence)	Axial, Para-Sagittal	3.8/1.7	0.5 × 0.5 × 4.0	250

**Table 2:** Results of the univariate analysis comparing patients with and without substantial LVSI on definitive histological specimen. The myometrial invasion was classified as 0: no myometrial invasion, 1: <50% of myometrial invasion, 2: >50% myometrial invasion. Significant predictors of LVSI are bolded.

Variable		NO LVSI (n = 179)	Substantial LVSI (n = 66)	*p*
Aggressive histological subtype at biopsy		44 (24.6%)	30 (45.5%)	**0.0016**
Presence of myometrial invasion on MRI		139 (77.7%)	65 (98.5%)	**0.0001**
Presence of myometrial infiltration exceeding 50% on MRI		43 (27.7%)	43 (66.2%)	**<0.0001**
EC/uterus on MRI (median)		0.03 (IQR: 0.01–0.088)	0.16 (IQR: 0.1–0.3)	**<0.0001**
Infiltration depth on MRI	0	39 (21.8%)	1 (1.5%)	**<0.0001**
	1	97 (54.2%)	22 (33.3%)	
	2	43 (24%)	43 (65.2%)	
Minimal tumor-to-serosa distance on MRI (median, mm)		6.0 (IQR: 3.0–9.0)	3.0 (2.0–5.0)	**<0.0001**
Serosal or subserosal involvement on MRI		2 (1.1%)	11 (16.7%)	**<0.0001**
Tubaric or adnexal involvement on MRI		3 (1.7%)	2 (3%)	0.5068
Cervical stromal invasion on MRI		6 (3.4%)	19 (28.8%)	**<0.0001**
Parametrial involvement on MRI		0 (0%)	5 (7.6%)	**0.0002**
Vaginal involvement on MRI		0 (0%)	3 (4.5%)	**0.0042**
Pelvic nodal involvement on MRI		5 (2.8%)	16 (24.2%)	**<0.0001**
Lumbar nodal involvement on MRI		0 (0%)	2 (3%)	**0.0196**
Pelvic peritoneal carcinosis		2 (1.1%)	1 (1.5%)	0.821

**Table 3:** Results of the univariate analysis comparing MRI parameters associated with either aggressive or non-aggressive tumor histotypes. Histological subtype and tumor grade from the preoperative endometrial biopsy are also reported. Significant predictors of tumor aggressiveness are bolded.

Variable		Non-Aggressive Histotype (n = 159)	Aggressive Histotype (n = 86)	*p*
Histology type at biopsy	Endometroid	156 (98.1%)	51 (59.3%)	**<0.0001**
	Non Endometroid	3 (1.9%)	35 (40.7%)	
Histology grade at biopsy	1	86 (54.1%)	8 (9.3%)	**<0.0001**
	2	65 (40.9%)	16 (18.6%)	
	3	8 (5%)	62 (72.1)	
Aggressive histological subtype at biopsy		10 (6.3%)	64 (74.4%)	**<0.0001**
Presence of myometrial invasion on MRI		125 (78.6%)	79 (91.9%)	**0.0082**
Presence of myometrial infiltration exceeding 50% of its thickness on MRI		45 (32.8%)	41 (49.4%)	**0.015**
EC/uterus (median)		0.03 (IQR: 0.01–0.098)	0.11 (IQR: 0.1–0.28)	**<0.0001**
Infiltration depth MRI	0	33 (20.8%)	7 (8.1%)	**0.0026**
	1	81 (50.9%)	38 (44.2%)	
	2	45 (28.3%)	41 (47.7%)	
Minimal tumor-to-serosa distance on MRI (median, mm)		6.0 (IQR: 3.0–9.0)	4.0 (2.0–6.0)	**0.0003**
Serosal or subserosal involvement on MRI		2 (1.3%)	11 (12.8%)	**0.0001**
Tubaric or adnexal involvement on MRI		2 (1.3%)	3 (3.5%)	0.239
Cervical stromal invasion on MRI		9 (5.7%)	16 (18.6%)	**0.0014**
Parametrial involvement on MRI		0 (0%)	5 (5.8%)	**0.0022**
Vaginal involvement on MRI		0 (0%)	3 (3.5%)	**0.018**
Pelvic nodal involvement on MRI		7 (4.4%)	14 (16.3%)	**0.0016**
Lumbar nodal involvement on MRI		0 (0%)	2 (2.3%)	0.054
Pelvic peritoneal carcinosis on MRI		2 (1.3%)	1 (1.2%)	0.9486

## Data Availability

Data are unavailable due to privacy restrictions.

## Abbreviations

The following abbreviations are used in this manuscript:

MRI	Magnetic Resonance Imaging
EC	Endometrial Cancer
FIGO	International Federation of Gynecology and Obstetrics
LVSI	Lymphovascular Space Invasion
